# Targeting of radiolabeled J591 antibody to PSMA-expressing tumors: optimization of imaging and therapy based on non-linear compartmental modeling

**DOI:** 10.1186/s13550-016-0164-0

**Published:** 2016-01-22

**Authors:** Edward K. Fung, Sarah M. Cheal, Shoaib B. Fareedy, Blesida Punzalan, Volkan Beylergil, Jawaria Amir, Sandhya Chalasani, Wolfgang A. Weber, Daniel E. Spratt, Darren R. Veach, Neil H. Bander, Steven M. Larson, Pat B. Zanzonico, Joseph R. Osborne

**Affiliations:** Department of Medical Physics, Memorial Sloan Kettering Cancer Center, 1275 York Avenue, New York, NY 10065 USA; Department of Radiology, Memorial Sloan Kettering Cancer Center, 1275 York Avenue, New York, NY 10065 USA; Molecular Pharmacology and Chemistry Program, Memorial Sloan Kettering Cancer Center, 1275 York Avenue, New York, NY 10065 USA; Molecular Imaging and Therapy Service, Department of Radiology, Memorial Sloan Kettering Cancer Center, 1275 York Avenue, New York, NY 10065 USA; Department of Medicine, Weill Medical College of Cornell University, 1300 York Avenue, New York, NY 10065 USA; Department of Radiation Oncology, Memorial Sloan Kettering Cancer Center, 1275 York Avenue, New York, NY 10065 USA

**Keywords:** J591, PSMA, ImmunoPET, Zirconium-89, Iodine-124, Non-linear kinetic model

## Abstract

**Background:**

We applied a non-linear immunokinetic model to quantitatively compare absolute antibody uptake and turnover in subcutaneous LNCaP human prostate cancer (PCa) xenografts of two radiolabeled forms of the humanized anti-prostate-specific membrane antigen (PSMA) monoclonal antibody J591 (^124^I-J591 and ^89^Zr-J591). Using the model, we examined the impact of dose on the tumor and plasma positron emission tomography (PET)-derived time-activity curves. We also sought to predict the optimal targeting index (ratio of integrated-tumor-to-integrated-plasma activity concentrations) for radioimmunotherapy.

**Methods:**

The equilibrium rates of antibody internalization and turnover in the tumors were derived from PET images up to 96 h post-injection using compartmental modeling with a non-linear transfer rate. In addition, we serially imaged groups of LNCaP tumor-bearing mice injected with ^89^Zr-J591 antibody doses ranging from antigen subsaturating to saturating to examine the suitability of using a non-linear approach and derived the time-integrated concentration (in μM∙hours) of administered tracer in tumor as a function of the administered dose of antibody.

**Results:**

The comparison of ^124^I-J591 and ^89^Zr-J591 yielded similar model-derived values of the total antigen concentration and internalization rate. The association equilibrium constant (*k*_a_) was twofold higher for ^124^I, but there was a ~tenfold greater tumoral efflux rate of ^124^I from tumor compared to that of ^89^Zr. Plots of surface-bound and internalized radiotracers indicate similar behavior up to 24 h p.i. for both ^124^I-J591 and ^89^Zr-J591, with the effect of differential clearance rates becoming apparent after about 35 h p.i. Estimates of J591/PSMA complex turnover were 3.9–90.5 × 10^12^ (for doses from 60 to 240 μg) molecules per hour per gram of tumor (20 % of receptors internalized per hour).

**Conclusions:**

Using quantitative compartmental model methods, surface binding and internalization rates were shown to be similar for both ^124^I-J591 and ^89^Zr-J591 forms, as expected. The large difference in clearance rates of the radioactivity from the tumor is likely due to differential trapping of residualizing zirconium versus non-residualizing iodine. Our non-linear model was found to be superior to a conventional linear model. This finding and the calculated activity persistence time in tumor have important implications for radioimmunotherapy and other antibody-based therapies in patients.

## Background

Prostate-specific membrane antigen (PSMA) is an integral cell membrane glycoprotein (~100 kDa) that is present on the surface of epithelial prostate cancer (PCa) cells [1]. While PSMA is expressed in normal human prostate cells and certain other normal tissues, it is highly upregulated in PCa, making it a promising diagnostic and therapeutic target [2]. A variety of monoclonal antibodies (mAbs) specific for intracellular and extracellular epitopes of PSMA have been prepared [3–6], with two variants in particular demonstrating high (low nM) affinity and specific and efficient targeting in vivo: the murine mAb 7E11, which binds an intracellular domain of PSMA, and the humanized mAb J591, which binds to an extracellular domain of PSMA. 7E11 has been investigated clinically as a SPECT imaging agent for recurrent and metastatic PCa [7] (^111^In-7E11, ProstaScint™) as well as therapeutically as the Y-90 conjugate [8]. Like 7E11, J591 has also been clinically investigated in a variety of radiolabeled forms for both positron emission tomography (PET) imaging (e.g., in the form of ^89^Zr-J591) [9] as well as therapy (e.g., as ^177^Lu-J591) [10]. When J591 binds to the external domain of PSMA on LNCaP prostate cancer cells, the J591/PSMA complex undergoes endocytosis via clathrin-coated pits and accumulates in endosomes (3). Indeed, the surface binding and subsequent internalization are the fundamental rationale for using J591 as a carrier for residualizing radiometals and cytotoxic drug conjugates [11–13] that rely on such internalization to maximize their therapeutic effectiveness.

Quantitative imaging and kinetic analysis of a theranostic agent allows for collection of patient-specific information regarding optimization of a subsequently administered radioimmunotherapeutic or antibody-drug conjugate. This is an emerging paradigm recently demonstrated by Zanzonico et al. in colorectal patients administered with radiolabeled anti-A33 humanized mAb and undergoing serial PET imaging and blood sampling [14]. Collectively, such data can be used to derive estimates of radiation doses or toxin concentrations in tumor and normal tissues to assess the risk/benefit of a particular treatment as well as to calculate an optimum mAb dose for the treatment. This is a practical and important step towards a personalized precision medicine approach: the tailoring of antibody-vector dose to achieve the highest concentration at antigen-positive sites while sparing normal tissue.

The effective implementation of a theranostic and/or a companion diagnostic requires that the radiotracer, ideally, should provide quantitative readouts of the antigen density and other antigen-antibody binding parameters. This is because therapeutic efficacy is, in part, correlated with magnitude and duration of receptor occupancy by the drug. To achieve this without interfering with subsequent drug delivery, administration of subsaturating doses of tracer can be used to estimate total receptor occupancy, while accounting for differences in affinity between the naked antibody and the antibody-conjugate (i.e., the diagnostic and the therapeutic reagents, respectively). In addition, initial estimates of other pertinent parameters such as the antigen density per cell and the apparent affinities of the naked antibody and antibody-conjugate can be determined using standard in vitro assays.

The aim of this study was to examine the binding kinetics of J591 in a murine xenograft model of PCa using quantitative PET and compartmental analysis of two positron-emitting forms of this antibody radiolabeled with either ^89^Zr or ^124^I. It is generally known that these two isotopes have different fates following internalization. The free radiometal ^89^Zr is trapped (or residualized) in the cell while the free iodide generally exits the cell rather quickly following such internalization of the antibody construct [15–19]. These differences could have implications for image quality and kinetics (e.g., see [20] for intra-patient comparison of an anti-CAIX antibody radiolabeled with either ^131^I or ^111^In).

**Fig. 1:**
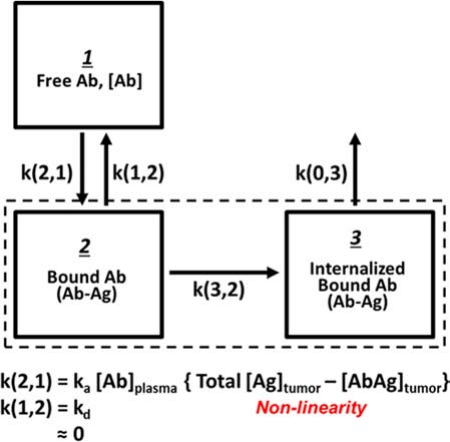
Schematic diagram of the non-linear compartmental model developed to represent saturable antibody kinetics. General notation: *k*(*a*,*b*) indicates flow to compartment *a* from compartment *b*

Governed by the law of mass action, at higher mAb doses, the amount of free antigen available for binding may be decreased to such an extent that the association rate of antibody is demonstrably affected [21]. To extrapolate high-dose antibody residence at the tumor from low-dose estimates of binding parameters, the model must be able to account for decreased concentration of available antigen as a function of dose [22]. To this end, we introduced a non-linear association term into the compartment model to represent bimolecular antigen-antibody binding kinetics (Fig. 1) [23]. This modification would be crucial in planning and optimizing therapeutic doses based on low-dose diagnostic data.

## Methods

### Reagents, antibodies, and cells

The humanized mAb J591 was kindly provided by Dr. Neil Bander (Weill Medical College of Cornell University, New York, NY). The PSMA-positive LNCaP cell line was obtained from American Type Culture Collection and maintained in culture by serial passage using standard conditions: RPMI 1640 supplemented with 10 % FCS at 37 °C in an environment containing 5 % CO_2_. The antigen density *B*_max_ and dissociation equilibrium constant *K*_d_ for J591 and intact (viable) LNCaP cells were previously determined using in vitro saturation binding assays and reported to be 600,000–800,000 sites/cell and 1.83 ± 1.21 nM, respectively [24].

### Radiolabeled mAbs

The positron-emitting starting reagents [^124^I]NaI (*t*_1/2_ = 4.18 days) and [^89^Zr]Zr-oxalate (*t*_1/2_ = 3.17 days) were provided by the Memorial Sloan Kettering Radiochemistry & Molecular Imaging Probes Core Facility. Activity measurements were made either with a dose calibrator (Capintec) or an automated gamma counter (PerkinElmer Wizard 3). For ^89^Zr radiolabeling, J591 was conjugated with isothiocyanatobenzyl-desferrioxamine (SCN-DFO; Macrocyclics, Dallas, TX) and subsequently radiolabeled with ^89^Zr-oxalate as described [9] (specific activity (SA) 118 MBq/mg; radiolabeling efficiency (RE) 70 %; minimum immunoreactivity (IR) 90 %). J591 was radiolabeled with ^124^I as previously described [24] using the IODOGEN method to a SA of 213 MBq/mg (RE 60 %; IR 75 %). The radiochemical purity of each tracer was determined using either instant thin-layer chromatography with 5 mM DTPA pH 5.0 or 10 % trichloroacetic acid as the elution solvent for ^89^Zr-mAb or ^124^I-mAb, respectively, and was routinely >98 %. To prepare the different mAb mass doses, non-radioactive J591 was added as necessary to achieve the final desired mAb mass after drawing up the ^89^Zr-J591 [155-243 μCi (5.7–9.0 MBq) per animal] or ^124^I-J591 [171-267 μCi (6.3–9.9 MBq) per animal], based on the initial SA of the respective preparations.

### Xenograft model

All animal experiments were approved by the Institutional Animal Care and Use Committee of Memorial Sloan Kettering Cancer Center (MSKCC), and institutional guidelines for the proper and humane use of animals in research were followed. Male athymic nude mice (outbred, 4–6 weeks old) were obtained from Harlan Laboratories (Indianapolis, IN) and allowed to acclimate in the MSKCC vivarium for at least 1 week prior to use. For tumor inoculation, cultured LNCaP cells were trypsinized using a solution of 0.25 % trypsin/0.05 % EDTA in Hank’s balanced salt solution without calcium or magnesium and prepared for subcutaneous (s.c.) injection in the lower flank as a suspension of five million cells in a final volume of 200-μl volume as a 1:1 mixture of reconstituted basement membrane (BD Matrigel, Collaborative Biomedical Products) and media. Established tumors (100–700 mm^3^) were observed in 4–6 weeks. Tumors were measured using external vernier caliper measurements and assumed to be spherical for calculation of volume.

### PET imaging studies

All radiolabeled antibodies were administered intravenously (i.v.) via the tail vein into groups of mice (*n* = 2–5) bearing s.c. LNCaP tumors at *t* = 0 after gentle warming of each animal with a heat lamp. Prior to scanning, animals were anesthetized using an inhaled mixture of 1.5–2 % isofluorane (Baxter Healthcare, Deerfield, IL) and air and placed on either the microPET Focus 120 (Concorde Microsystems, Knoxville, TN) or Inveon PET/CT (Siemens Medical Solutions) small animal scanner. No blocking with cold iodide was performed in mice given ^124^I-J591.

For comparison of ^89^Zr- and ^124^I-J591, a total of four groups were imaged. For imaging with ^89^Zr-J591, two different mAb-carrier mass levels (expressed as micrograms of mAb) were studied: 60 (*n* = 3) or 180 (*n* = 2) (0.4 and 1.2 nmol, respectively). For imaging with ^124^I-J591, groups were injected with either 45 (*n* = 5) or 180 (*n* = 2) (0.3 and 1.2 nmol, respectively). All groups were imaged at least five time points from 4 to 96 h p.i.

For compartmental analysis of mAb-carrier dose escalation with ^89^Zr-J591 as the tracer, additional imaging studies were performed with five different dose levels (micrograms of mAb): 58 (*n* = 4), 180 (*n* = 4), 240 (*n* = 4), 1000 (*n* = 5), and 2000 (*n* = 3) (0.39, 1.20, 1.60, 6.67, and 13.3 nmol, respectively). All groups were imaged at 24, 48, and 96 h p.i.

PET data were acquired using an energy window of 350–700 keV and a coincidence timing window of 6 ns. The list mode data were sorted into two-dimensional histograms by Fourier rebinning, and image reconstruction was performed by filtered back projection with a 128 × 128 × 63 (0.72 × 0.72 × 1.33 mm) matrix. The final data were parameterized (as the percent of theinjected dose per gram of tissue (%ID/g)) by first converting the voxel-counting rates to activity concentrations using empirically determined calibration factors for the specific isotope, followed by decay correction to the time of injection and normalization to the administered activity. No attenuation or scatter was applied. Tumor and heart were manually delineated in 3D reconstructed images of each mouse, and time-activity curves (TACs) were derived from the regions of interest (ROI) using ASIPro VM™ software (Concorde Microsystems). TAC values were corrected for partial volume effects based on tumor size. The heart TAC was utilized as the blood input function for fully quantitative kinetic modeling.

### Compartmental modeling

A non-linear compartmental model was developed and implemented using SAAM II modeling software (v. 1.1.1, SAAM Institute, University of Washington, WA). This model (Fig. 1) includes two tissue compartments and a blood compartment representing the plasma input, *q*1. The two tissue compartments modeled are the cell surface binding compartment, *q*2, and an intracellular space compartment where internalized antibody resides, *q*3. Unidirectional flow is modeled among all three compartments. The parameter *k*_2,1_ represents the flow from the plasma compartment and binding to surface antigen. The parameter *k*_3,2_ represents the passage of the antigen-antibody (Ag-Ab) complex from the surface into the cell. Finally, the fate of the radiolabel, whether sequestered in the cell or removed from the tumor, is modeled by the parameter, *k*_0,3,_ which represents efflux from the intracellular compartment and thus the tumor overall. Reverse rates from the surface back to arterial plasma and from the internalized compartment back to the surface are excluded. These are based on the following assumptions: (1) there is negligible dissociation of the antibody after binding to the cell surface antigen; (2) once the Ag-Ab complex has been internalized, it cannot be returned to the surface in the intact radiolabeled form; and (3) separation of the radiolabel from the Ag-Ab complex only occurs after internalization.

Binding of radiolabeled antibody to surface antigen is modeled as a non-linear saturable process given by the equation for *k*_2,1_. This binding rate is dependent on the concentrations of unbound and non-internalized antigen present on the cell surface, specifically, the difference ([Ag_total_] − [Ag-Ab]), where [Ag_total_] represents the estimated total antigen presented at the surface (i.e., *B*_max_) and [Ag-Ab] represents the concentration of bound Ag-Ab complex. The [Ag-Ab] value changes with time and is derived from the measured tumor activity. The blood component of the tumor activity is assumed to be 8 % of the measured plasma activity. A similar model was previously described by Cheal et al. [23]. The input function was measured directly from the serial PET images by drawing an ROI over the heart (i.e., the left ventricle). Starting values for the fitted parameters were derived from in vitro data [24, 25]. Data points were Poisson-weighted in fitting with SAAM II.

Model parameters derived from the low-dose 60 μg ^89^Zr-J591 TACs in combination with image-derived input functions from the higher dose (180 and 240 μg) were used to simulate higher-dose tumor TACs. These were compared to the measured TACs at the higher-dose levels. TACs for higher-dose levels were also simulated using a conventional linear compartmental model fit to the low-dose measurements. The linear model was identical to the non-linear model described here with the exception that the antigen-antibody association rate is independent of [Ag-Ab].

## Results

### Serial PET images and TAC curves

**Fig. 2:**
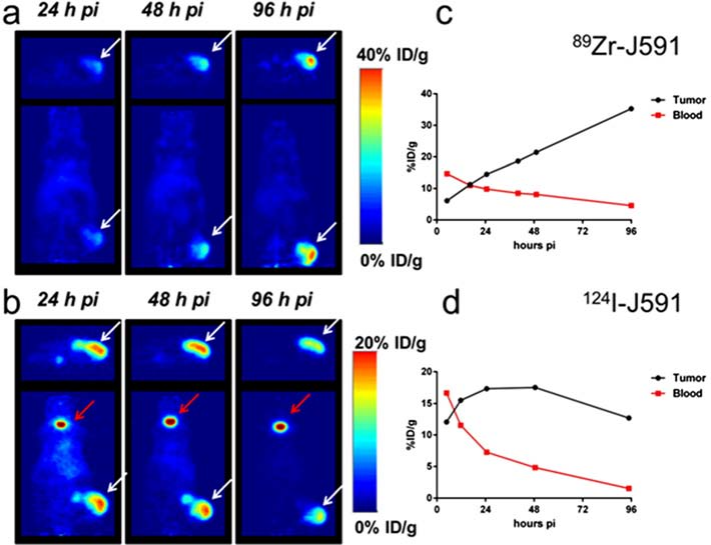
Representative serial PET images at 24, 48, and 96 h p.i. of mice bearing s.c. LNCaP tumors imaged with either ^89^Zr-J591 (**a**) or ^124^I-J591 (**b**). The tumor is indicated in all of the transverse and coronal images by a *white arrow*. The thyroid is indicated by the *red arrow* in the coronal ^124^I-J591 images only. Time-activity curves for tumor and blood corresponding to the images in **a** and **b** of ^89^Zr- (**c**) and ^124^I-J591 (**d**) imaged mice

Representative serial planar images for both tracers (administered mass of mAb 180 μg) at 24, 48, and 96 h p.i. are shown in Fig. 2 (left). There is sufficient contrast for both the ^124^I- and ^89^Zr-labeled antibody as early as 24 h p.i to allow clear delineation of the s.c. LNCaP tumors present in the lower flank of the mice. The estimated average tumor-to-blood activity concentration ratios for the ^89^Zr- and ^124^I-J591 at 24 h p.i are 1.5 and 2.4, respectively. At 96 h p.i., the average tumor-to-blood ratios increased to approximately 7.7 and 8.5 for ^89^Zr- and ^124^I-J591, respectively. TACs for plasma and tumor for each tracer are provided in Fig. 2 (right). There are major differences in the tumor TAC between the tracers, suggesting a difference in the in vivo biological fate of the two PET isotopes. For ^89^Zr-J591, the tumor activity continuously increased from 24 to 96 h p.i., rising from ~15 to ~40 %ID/g, respectively. Conversely, tumor activity concentration for ^124^I-J591 reaches a maximum uptake in tumor of ~18 %ID/g at 24 h p.i. which persisted for the following 24 h before dropping to ~12.5 %ID/g at 96 h p.i. Furthermore, the thyroid is clearly visible in the ^124^I-J591 images, indicating that significant de-iodination occurred in vivo. These characteristics of radiolabeled J591 tracer in mice carrying s.c. LNCaP are similar to those in our previously published reports for ^89^Zr-DFO-J591 [26] and ^131^I-J591 [27] investigated in the same mouse model.

### Comparison of ^89^Zr- and ^124^I-J591 PET kinetic model parameter estimates

**Fig. 3:**
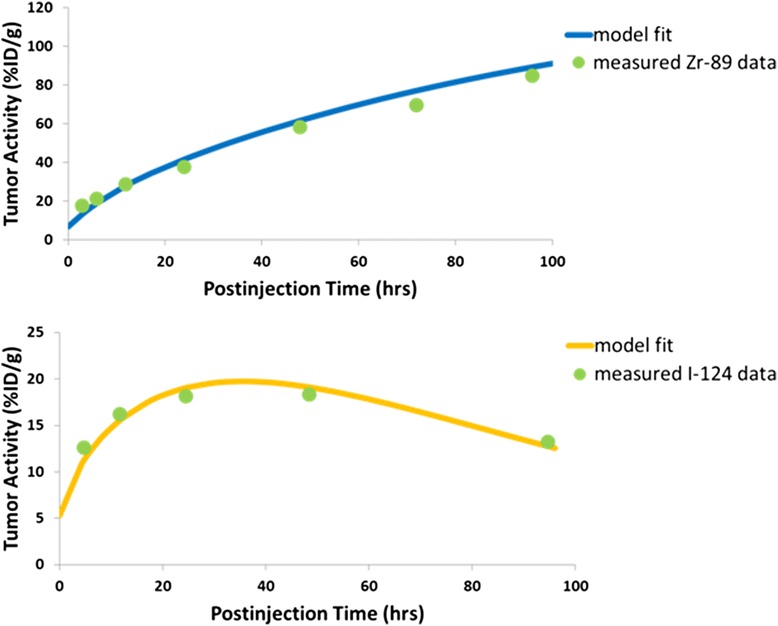
Representative data for 60-μg dose of ^89^Zr-J591 (*top*) and 180-μg dose of ^124^I-J591 (*bottom*). Model-fitted TACs shown over measured experimental data

Estimates of four different kinetic model parameters were derived for the two different tracers: total LNCaP PSMA antigen concentration (Ag_total_), association rate constant (*k*_a_), internalization rate (*k*_3,2_), and efflux rate (*k*_0.3_). Parameter estimates are provided as average ± standard deviation with their appropriate units for the ^124^I (*n* = 7) and ^89^Zr (*n* = 5) groups. Representative TACs generated from model fits are shown overlayed on the relevant measured data in Fig. 3. Root-mean-squared error calculated for the fitted TACs ranged from 0.31 to 1.14 %ID/g for ^124^I-J591 and from 1.06 to 7.18 %ID/g for ^89^Zr-J591. The [Ag_total_] was found to be almost identical for both radiolabeled antibodies (^124^I 1.3 ± 0.2 μM, ^89^Zr 1.2 ± 0.2 μM). This result was expected, as there were no apparent differences in *B*_max_ between the two tracers in vitro. These values are also consistent with previously published in vitro results, which predict an antigen concentration of between 1.00 and 1.33 μM [24], and serve to validate in part our modeling process. The tumor xenografts were within the same volume range for all groups of animals. Secondly, although the association rate constant (*k*_a_) was also found to be significantly higher (*p* < 0.05) for ^124^I- than for ^89^Zr-J591, with values of 7.0 × 10^7^ ± 1.5 × 10^7^ and 4.4 × 10^7^ ± 1.9 × 10^7^ M^−1^h^−1^, respectively, the magnitude of the difference is less than twofold. We believe this indicates that uptake dynamics were not drastically affected by the choice of radiolabel. Thirdly, the internalization kinetics were compared and the difference between internalization rates was not significant (^124^I 242 ± 125 M^−1^h^−1^, ^89^Zr 270 ± 148 M^−1^h^−1^). For the 180-μg-dose data, the average maximum internalization rate is 1.2 × 10^13^ molecules per gram of tissue per hour. Using an estimate of 10^8^ LNCaP cells per gram, we calculate that this is equal to 1.2 × 10^5^ molecules internalized per cell per hour or 20 % of the estimated 600,000 sites per cell. This is in agreement with our hypothesis that the antibody delivery mechanism can be loaded with different cargo types yet retain the same delivery kinetics.

**Fig. 4:**
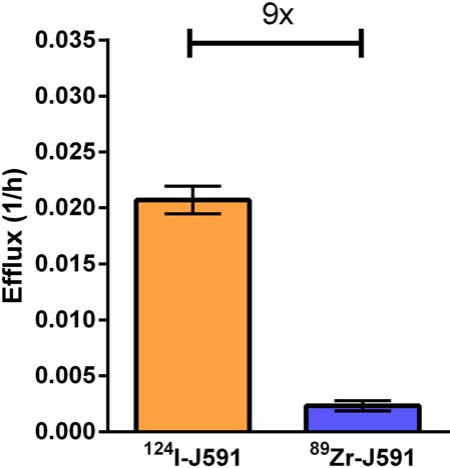
Comparison of efflux rates for ^124^I- and ^89^Zr-labeled antibody (shown as average ± standard error of the mean; *n* = 7 and *n* = 5, respectively). There is an approximately ninefold difference in rates for the two radiolabeled forms

Finally, the parameter with the greatest difference between the iodine- and zirconium-labeled experiments was the efflux value, with ^124^I-J591 having a very significant ninefold higher efflux rate of the radiolabel than that of ^89^Zr-J591 (*p* < 0.0000005) (Fig. 4).

### Estimation of accumulated activity in separate tumor compartments and targeting index

**Fig. 5:**
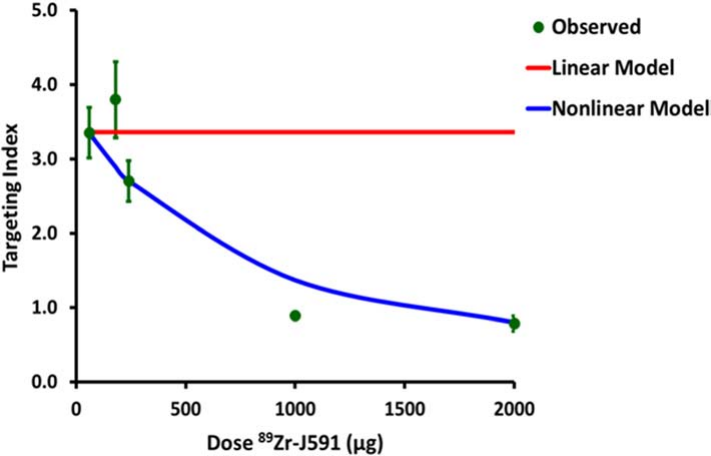
Targeting index defined as ratio of integral of activity at tumor to integral of activity in plasma as a function of dose. Projected targeting indices based on low-dose parameter estimates are shown for both our non-linear Ag-Ab binding model and for a conventional linear model

From the fitted tumor and plasma TACs, the time integrals of the respective activity concentrations were calculated. The targeting index was defined as the ratio of the time-integrated activity concentration in the tumor to the time-integrated activity concentration in plasma. This serves as a metric of the exposure of the tumor to a therapeutic radionuclide or drug versus non-tumor systemic exposure. Targeting indices were calculated from the observed data and plotted against the administered antibody dose, indicating that the targeting index is much lower at higher antibody doses. Using “best-fit” parameters derived from kinetic modeling of tracer ^89^Zr-J591 (i.e., 60 μg/mouse), the TACs for higher doses up to 2000 μg of ^89^Zr-J591 were obtained to determine the correlation between predicted TACs generated via kinetic modeling and empirically measured TACs. The targeting indices calculated using curves generated with the non-linear compartment model are shown in Fig. 5 and show a very close concordance with the observed data. The maximum targeting index for the experimental Ab levels was 3.4 at the lowest dose of 60 μg. Predicted targeting index at a 2000-μg dose of ^89^Zr-J591 was 0.80 compared to the observed targeting index for that dose of 0.78 ± 0.10 (mean ± standard error). Analogous predicted TACs generated using a compartment model with a linear unimolecular binding component (i.e., not dependent on antigen concentration) were also generated. Corresponding targeting indices were calculated for the higher doses based on the assumption of a linear Ag-Ab binding component. As can be seen, the predicted targeting indices using a conventional linear model, particularly for the very high saturating or near-saturating doses, do not agree well with the observed data. The linear model here predicts a constant, dose-independent targeting index. The antigenic sites are saturated at high antibody doses, and further increases in the administered dose do not produce corresponding increases in targeting index. Rather, the reverse is true, with higher doses generally yielding lower targeting indices.

**Fig. 6:**
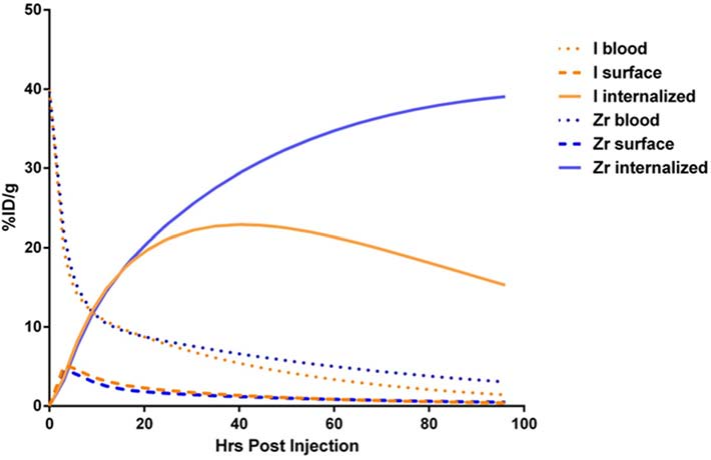
Simulated rate of surface binding and internalization for 180-μg Ab doses of ^124^I- and ^89^Zr-J591. Surface binding decreases with time, but activity detected at tumor remains high due to internalized Ab-Ag. Differences in total observed tumor activity can be explained primarily by differences in the internalized portion of injected dose while surface binding curves remain nearly identical across radiolabels

The compartmental model separates uptake into surface and internalized compartments. Internalized activity can thus be extracted from the total tumor imaging signal. In Fig. 6, simulated internalized activities using actual plasma TACs and fitted model parameters are shown for both the iodine- and zirconium-labeled antibodies. Early time points show similarity in the activity in the internalized compartment for both radiolabeled forms of J591. The difference in internalized compartment activity does not exceed 1 %ID/g until about 24 h p.i. As can be seen, due to the residualizing nature of the radiometal, activity in the internalized compartment persists at a high level relative to that for iodinated antibody. ^124^I-J591 shows signs of efflux of activity from the internalized compartment with activity peaking at 40.5 h p.i. At 96 h p.i., activity in the internalized compartment for ^89^Zr-J591 is predicted to be approximately 2.7 times higher than that for ^124^I-J591.

## Discussion

Two different radiolabeled forms of the same mAb were injected into groups of LNCaP tumor-bearing mice and their tumor uptake kinetics compared by serial PET imaging: J591, a specific PSMA-targeted antibody that is known to be internalized after binding to extracellular antigen, labeled with either ^89^Zr, a residualizing PET isotope, or ^124^I, a non-residualizing PET isotope.

This work demonstrates the development and use of a non-linear compartment model to quantitate the in vivo characteristics of an administered radiolabeled antibody in a pre-clinical murine cancer therapy trial as a function of the antibody dose. Values for biologically relevant parameters, namely, antibody-antigen association rate, total antigen concentration in tumor, internalization rate, and clearance rate from the tumor, were estimated using the model. Serial PET data from multiple scans repeated on the same animals up to 96 h p.i. were successfully fit to the proposed model and demonstrated a marked difference in clearance for the two radioisotopes used. This will inform future development of radioimmunotherapy based on this antibody model; for example, estimation of optimum Ab-administered mass on a patient-specific basis during treatment planning. The model also enabled the evaluation of the “residence” of the radioisotope in the tumor, particularly in the internalized compartment. Quantitative imaging in this pre-clinical model has proven to be a useful tool in evaluating both the targeting and residence of radioimmunotherapy agents and thus their predicted efficacy while still in a pre-clinical phase.

Binding and internalization of J591 to PSMA-expressing tumor xenografts were similar when radiolabeled with either ^89^Zr or ^124^I. The similarity in binding parameters for these two radioisotopes suggests that radioisotope selection does not affect the delivery of the antibody to target cells. The similar binding and internalization rates support the concept of antibody as a targeting vehicle to which different payloads can be attached. Using this paradigm, an antibody system could be investigated and characterized in vivo using a positron-emitting radioisotope payload and PET imaging. An alternative radioisotope more suitable for therapy could then be substituted. While the in vivo fate of the particular payload (e.g., a different radioisotope) would still have to be characterized experimentally, we may not need to re-evaluate specifically the delivery and uptake kinetics of the antibody. This could potentially be extended to antibody-drug conjugates as well; additional experiments would be warranted to determine if the compartmental model were accurate for a particular antibody-drug conjugate (e.g., by comparing the cytotoxic drug residence to that of a surrogate radioisotope).

The difference in efflux of radioactivity from tumor between the two radiolabeled forms of mAb may be attributable to differential biological fate of the radioisotopes intracellularly. The large difference in the efflux rate is consistent with residualization of zirconium, but not of the iodine, in the target cells. Metal ions such as zirconium are known to be sequestered in cells. In this case, the identity of the zirconium-associated adduct that is sequestered has yet to be investigated. Iodine, on the other hand, is known not to be residualized and exits the cell and returns to the circulation much more rapidly. The appearance of the thyroid in the ^124^I antibody images is very likely attributable to the in vivo de-iodination of the antibody and physiologic avid accumulation of the resulting free iodine (i.e., iodide) in the thyroid.

With appropriate values estimated empirically for the free parameters, our model can be used to simulate tumor TACs under different conditions. We generated simulated TACs at the different dose levels for which we collected the mouse data. The non-linear model much more accurately predicted TACs at different dose levels than did a conventional (i.e., linear) model, which does not take into account the saturable nature of antigen-antibody binding. As previously stated, analysis of internalization and residence of activity at the tumor can have implications for radioimmunotherapy. In this case, the residualizing nature of radiometals resulted in zirconium remaining internalized and thus accumulating to higher activities for longer periods of time than did iodine. This directly affects the absorbed dose to the tumor, with potential radiobiological implications such as the proximity of the radionuclide to the radiosensitive cell nucleus. Also, estimation of the internalized fraction can be of significant importance to the prediction of efficacy of a particular antibody-drug conjugate dose, as the antibody-drug conjugate is frequently administered in a pro-drug form and activated upon internalization. The concentration of the drug in the internalized compartment may be more indicative of therapeutic efficacy than the total concentration in the tumor.

## Conclusions

We have successfully applied a non-linear compartment model to pre-clinical PET data of in vivo tumor uptake of a targeted antibody. Using the model, we have quantitatively assessed parameters of biological and clinical significance including antigen concentration, internalization rates, and efflux rates of antibody from tumor. We have demonstrated that the kinetics of two radiolabeled forms of the humanized mAb J591, ^124^I- and ^89^Zr-J591, do not differ drastically in terms of initial uptake. We have shown, however, that there is a large difference in residence time of the PET imaging signal, likely due to internalization and subsequent residualization of the ^89^Zr label versus rapid efflux of the ^124^I label. We found that our non-linear compartment model performed well in predicting the targeting index at higher doses and was far more appropriate than a linear compartment model. This has important implications for radioimmunotherapy dose optimization, especially when using lower doses or diagnostic surrogates for patient-specific dose planning.

## Ethics approval and consent to participate

Ethical approval (human prostate cancer xenografts): All procedures performed in studies involving human participants were in accordance with the ethical standards of the institutional and/or national research committee and with the 1964 Helsinki declaration and its later amendments or comparable ethical standards.

Ethical approval (mice): All animal experiments were approved by the Institutional Animal Care and Use Committee of MSKCC, and institutional guidelines for the proper and humane use of animals in research were followed. In addition, all applicable international and national guidelines for the care and use of animals were followed.

As this research used only prostate xenografts, informed consent did not apply to this study.
